# Reading out a spatiotemporal population code by imaging neighbouring parallel fibre axons *in vivo*

**DOI:** 10.1038/ncomms7464

**Published:** 2015-03-09

**Authors:** Christian D. Wilms, Michael Häusser

**Affiliations:** 1 Wolfson Institute for Biomedical Research and Department of Neuroscience, Physiology and Pharmacology, University College London, Gower Street, London WC1E 6BT, UK

## Abstract

The spatiotemporal pattern of synaptic inputs to the dendritic tree is crucial for synaptic integration and plasticity. However, it is not known if input patterns driven by sensory stimuli are structured or random. Here we investigate the spatial patterning of synaptic inputs by directly monitoring presynaptic activity in the intact mouse brain on the micron scale. Using *in vivo* calcium imaging of multiple neighbouring cerebellar parallel fibre axons, we find evidence for clustered patterns of axonal activity during sensory processing. The clustered parallel fibre input we observe is ideally suited for driving dendritic spikes, postsynaptic calcium signalling, and synaptic plasticity in downstream Purkinje cells, and is thus likely to be a major feature of cerebellar function during sensory processing.

## Abstract (web summary)

The spatiotemporal pattern of synaptic inputs is critical for synaptic integration and plasticity in neurons but whether these inputs are structured or random is not clear. Here the authors use *in vivo* calcium imaging to monitor the presynaptic activity of cerebellar parallel fibre axons and find clustered patterns of axonal activity during sensory processing.

## Graphical abstract

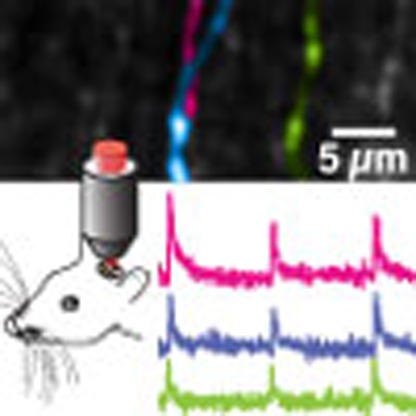

The spatial distribution of active synaptic inputs across the dendritic tree has a profound influence on the dynamics of synaptic integration and the induction of synaptic plasticity^1^,^2^. There is strong evidence from both experimental and modelling work that spatial clustering of active synaptic inputs represents an optimal stimulus for engaging a range of dendritic mechanisms. Clustered synaptic activity allows neurotransmitter uptake to be overwhelmed, enhancing pooling of spillover glutamate and engagement of high-affinity and extrasynaptic NMDA and metabotropic glutamate receptors^3^,^4^,^5^. Clustered synaptic input efficiently activates dendritic non-linearities, in particular dendritic spikes, triggering local calcium influx and enhancing the influence of dendritic inputs on somatic output^6^,^7^,^8^,^9^,^10^,^11^. Finally, focal synaptic input can trigger coordinated changes in synaptic strength at neighbouring spines^12^, and trigger compartmentalized changes in dendritic excitability^13^. These experimental findings have been bolstered by modelling work showing that experience-driven clustering of synaptic input is a powerful mechanism of memory storage^14^,^15^,^16^.

Despite the compelling evidence pointing to the importance of clustered inputs for dendritic function, very little is known, however, about the spatial structure of presynaptic activity engaged during sensory processing *in vivo*. Direct measurements of sensory-evoked calcium transients in dendritic spines *in vivo* have thus far failed to reveal clustering of activated synapses^17^,^18^. In contrast, there is indirect evidence that clustered inputs are activated by network activity^19^: imaging spontaneous spine calcium signals in organotypic hippocampal slices and in somatosensory cortex *in vivo* has revealed a tendency for active spines to be clustered^20^,^21^. Repetitive motor learning induces coordinated formation of clustered dendritic spines in layer 5 pyramidal cells *in vivo*^22^, consistent with enhancement of clustered synaptic inputs to dendrites following behavioural learning in the barn owl auditory system^23^. Finally, foraging behaviour triggers compartmentalized enhancement of dendritic excitability in a manner similar to clustered synaptic activation^24^. It remains unclear if these apparently contradictory results are due to methodological issues, or are related to differences in activity patterns in different neuronal circuits. Nevertheless, direct evidence for clustered activation of functional synaptic inputs *in vivo* has remained elusive.

The most direct way to map the spatial distribution of functional synaptic inputs is to measure activity across a population of presynaptic axons with sufficient spatial resolution to discriminate neighbouring axons during sensory stimulation. Here we have used two-photon imaging of axonal activity *in vivo*^25^,^26^ to map the distribution of active synaptic inputs on the micron scale in the molecular layer of the cerebellar cortex. This is a particularly attractive circuit for addressing this fundamental problem as the highly ordered anatomical structure of the cerebellar cortex allows readout of activity across a population of neighbouring parallel fibres (PFs), and makes it possible to directly relate this spatiotemporal pattern to the structure of the orthogonally oriented Purkinje cell dendritic tree. Moreover, classical models of cerebellar function^27^,^28^ provide testable predictions of PF activity.

Using *in vivo* two-photon imaging of cerebellar PFs, we can directly visualize the spatiotemporal patterns of activity in neighbouring axons during sensory stimulation. We find that parallel fibres active during sensory processing exhibit clustering on the micron scale. This clustering is expected to have a major impact on dendritic processing of PF inputs by the postsynaptic Purkinje cells.

## Results

### Imaging sensory-evoked activity in single PFs *in vivo*

**Figure 1:**
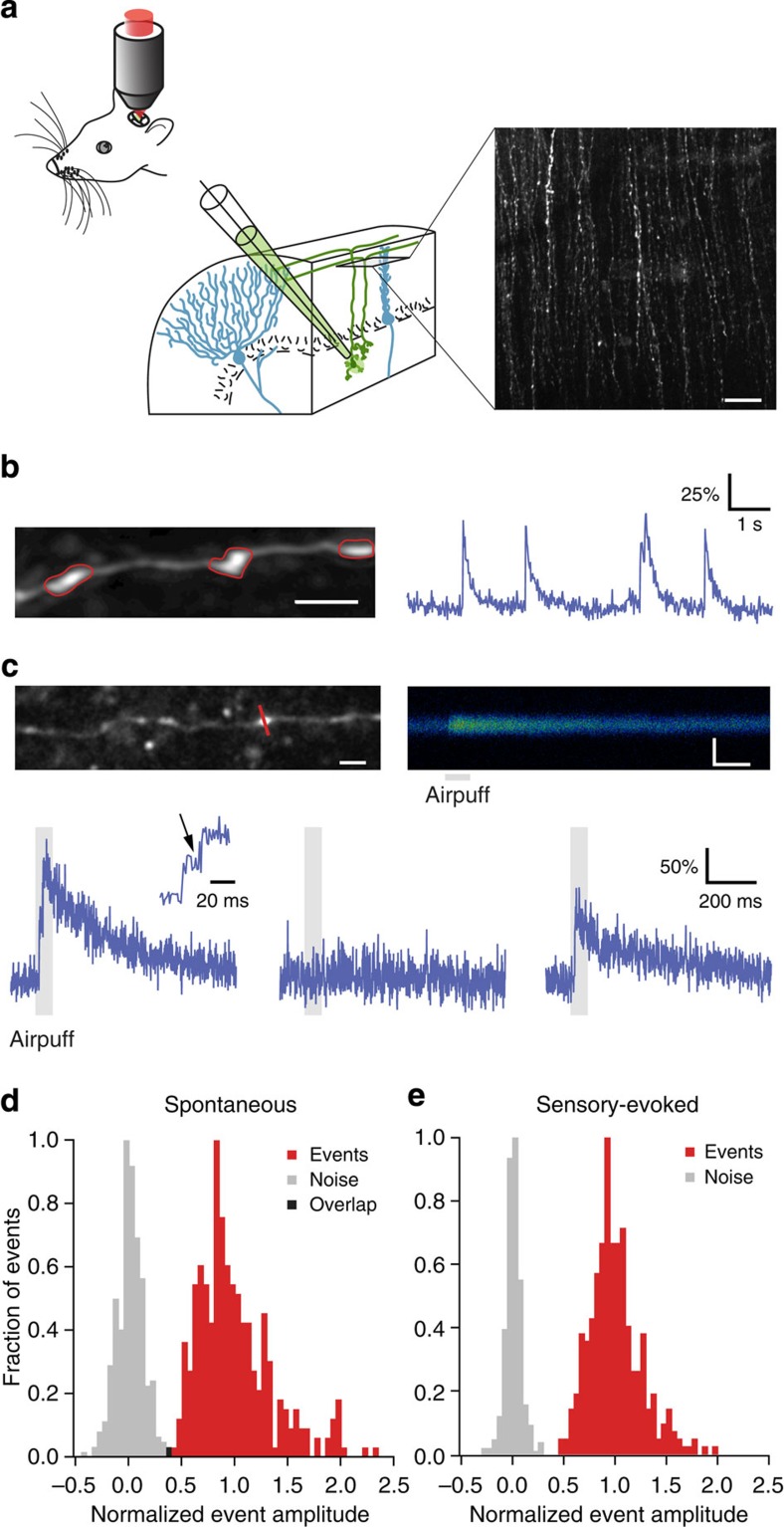
Imaging activity in single parallel fibres *in vivo*. (**a**) Schematic of the experimental configuration for two-photon imaging of activity in parallel fibres *in vivo*. Granule cells were electroporated with OGB-1, resulting in OGB-1-labelled parallel fibres, with single parallel fibre axons clearly visible in the molecular layer. Scale bar, 25 μm. (**b**) Spontaneous activity imaged in a single parallel fibre exhibiting three putative presynaptic boutons (red outlines). Right panel shows spontaneous calcium transients (extracted from the red-outlined pixels). Scale bar, 5 μm. (**c**) Line scan (red line in overview, top left; scale bar, 5 μm) across a putative presynaptic bouton reveals a sensory-evoked calcium transient (top right, vertical scale bar, 2 μm, horizontal scale bar, 100 ms). Three trials showing the variability of responses to sensory stimulation (grey bars) are shown below; note the shoulder in the rising phase of the first response (inset), and the clear difference between a failure (centre) and putative single AP response (right). (**d**,**e**) Signal-to-noise analysis of spontaneous (**d**) and sensory-evoked (**e**) events using amplitude histograms of events (red) and the corresponding noise (grey). Data pooled from multiple fibres (spontaneous: 343 events in 10 fibres from 5 animals; sensory evoked: 381 events in 8 fibres from 6 animals) by normalizing the amplitudes of all events from a given fibre to the respective mean response. As sensory-evoked responses were larger than spontaneous events, the noise peak of the ‘sensory-evoked’ panel appears narrower. Raw df/F_0_ values: spontaneous: noise=0.005±0.07, event=0.50±0.25; evoked: noise=0.005±0.06, event=0.88±0.32 (mean±s.d.).

We developed an approach for imaging the activity of single and multiple PF axons *in vivo* using two-photon microscopy. Granule cells in crus II of anaesthetized mice were loaded with the calcium indicator Oregon-Green-488-BAPTA-1 (OGB-1) using bulk electroporation^29^, allowing the activity of labelled PF axons to be visualized using *in vivo* two-photon imaging (Fig. 1a). Individual PF axons could be clearly resolved in the cerebellar molecular layer with sufficiently sparse labelling (Fig. 1b; percentage of labelled fibres=0.38±0.01% (mean±s.e.m.); *n*=11 regions from 9 mice). Large, all-or-none spontaneous Ca^2+^ transients, with kinetics and amplitudes consistent with action potential-dependent signals^30^,^31^ were observed in active axons spiking at a mean rate of 0.4±0.4 Hz (mean±s.d.; range 0.1–1.7 Hz; *n*=52 axons from 14 mice). This is consistent with the spontaneous spike rate of single-granule cells measured electrophysiologically *in vivo* under similar conditions (0.6±0.5 Hz; mean±s.d.; *n*=10 granule cells from 10 rats; *P*=0.16, Mann–Whitney test; combined data from refs 32,33). The majority of PFs appeared to be silent (19% and 0.5% of PFs showed spontaneous activity under ketamine-xylazine and isoflurane anaesthesia; *n*=8 and 6 mice, respectively). We verified that the silent fibres were capable of responding by activating them with a high [K^+^] solution (99% of fibres responded, *n*=78 fibres in 4 mice) or using electrical stimulation (91% of fibres responded, *n*=372 fibres in 5 mice).

To examine the responses to sensory stimulation, we used a brief sensory stimulus (70 ms airpuffs to the perioral region), which triggered reliable excitatory postsynaptic potentials and changes in spiking (both excitatory and inhibitory) in postsynaptic Purkinje cells (Supplementary Fig. 1 and Supplementary Note 1). This stimulation triggered Ca^2+^ transients in 2.8±0.6% (mean±s.e.m.; *n*=10 mice) of labelled PFs (Fig. 1c). These sensory-evoked calcium transients were present in all boutons over the full imaged length (up to 250 μm) of a responding PF, and were highly correlated across the boutons of a given fibre (Supplementary Fig. 2). Linescans (1 kHz line frequency) revealed that sensory-evoked calcium transients were composed of multiple events in at least 83% of PFs (Fig. 1c, Supplementary Fig. 3 and Supplementary Note 2). These sensory-evoked bursts typically consisted of two or three events during the rising phase of the transients (2.6±0.9; mean±s.d.; *n*=101 responses from 16 fibres in 12 mice) with a maximum frequency within the burst of 172±12 Hz (*n*=50 responses from 16 fibres in 12 mice). The sensory-evoked responses are comparable to the sensory-evoked bursts of spikes observed in single-granule cells *in vivo* using electrophysiological methods (2.6±0.9 spikes; mean±s.d.; *n*=10 GCs from 10 rats; *P*=0.61, *t*-test; combined data from refs 32,33). We find that response latencies are distributed bimodally, with sharp peaks at ~10 and ~22 ms, presumably corresponding to the trigeminal and somatosensory cortical projections^34^ (see Supplementary Fig. 3 and Supplementary Note 2). The signal-to-noise ratio (SNR) for the calcium transients was high for both spontaneous (SNR=6.9±0.07; mean±s.e.m.; *n*=337 events in 10 fibres from 5 mice; Fig. 1d) and evoked (SNR=8.5±0.25; mean±s.e.m.; *n*=340 events in 9 fibres from 6 mice; Fig. 1e) events. Taken together, these results indicate that our imaging approach can reliably detect both spontaneous and sensory-evoked activity in PFs.

### Evidence for sensory-evoked clustered activity in PFs

**Figure 2:**
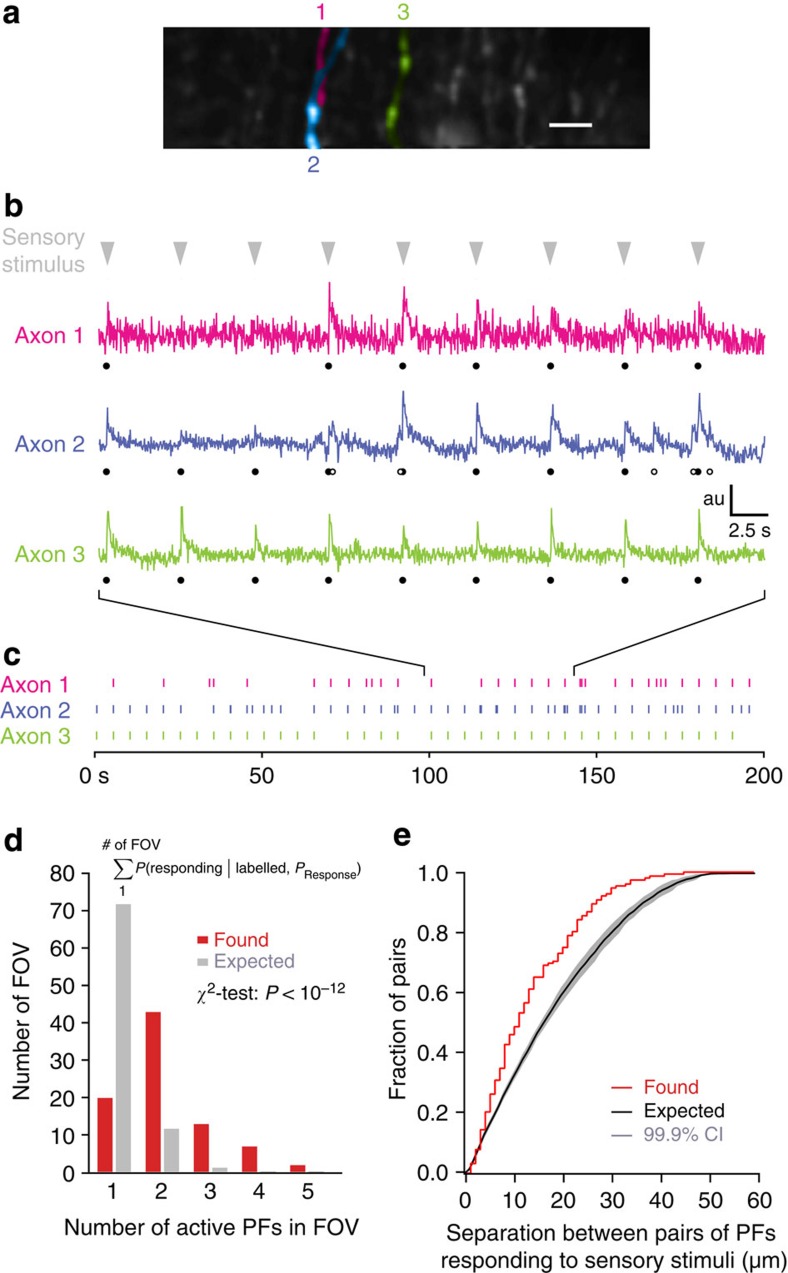
Sensory stimulation activates clusters of parallel fibres. (**a**) Image of parallel fibres labelled with OGB-1 *in vivo*; the three fibres responding to the sensory stimulus are colour coded. The separate identity of fibres 1 and 2 was verified using a three-dimensional high-resolution image. Scale bar, 5 μm. (**b**) Ca^2+^ signals from the responding parallel fibres during a train of sensory stimuli. Same colour code as in **a**, with stimulus timing indicated by the grey arrowheads. Stimulus-induced responses and spontaneous activity are indicated by filled and open circles, respectively. (**c**) Raster plot of events (40 airpuffs at 0.2 Hz). Note axons 1 and 2 (but not 3) also exhibit spontaneous events. (**d**) The number of fields of view containing 1–5 fibres exhibiting sensory-evoked responses from all experiments (red) are significantly different from the distribution expected (see equation) for randomly distributed responsive fibres (grey; *P*<10^−12^; *χ*^2^ test). (**e**) Cumulative histogram of the spatial separation between parallel fibres exhibiting sensory-evoked responses (red) compared with all pairs of labelled fibres from the same fields of view (black; average of 68 fields of view). The two distributions are significantly different (medians: active=11.0 μm, all=17.0 μm; *P*<10^−4^; Mann–Whitney test; *n*=68 fields of view) and the curve describing the experimental results is well separated from the 99.9% confidence interval (CI) around the expected distribution (grey area).

Next, we simultaneously imaged multiple neighbouring PFs in order to map the spatiotemporal pattern of PF activation on the micron scale. Activity was imaged across a population of labelled PFs during sensory stimulation using an imaging window (56 μm in the sagittal axis; Fig. 2a–c) that encompassed multiple labelled PFs (range 4–21 labelled PFs, median=13; *n*=85 fields of view in 28 mice). If PFs are activated independently of each other, the frequency of observing separate PFs responding to the same stimulus (‘co-active’ PFs) should follow a binomial distribution determined by the response probability of a single fibre (*P*_response_=0.028, see above) and the number of visible fibres in that imaging window. Instead, the observed distribution was significantly shifted towards higher numbers of axons (*P*<10^−13^; *χ*^2^-test, *n*=85 fields of view from 28 mice), indicating that neighbouring PFs tend to be co-active (Fig. 2d). Examination of the spatial separation of co-active PFs within a field of view showed that the inter-axon distance was significantly shorter than expected when compared with all pairs of labelled fibres from the same field of view (Fig. 2e; medians: active=11.0 μm, all=17.0 μm; *P*<10^−4^; Mann–Whitney test; *n*=68 fields of view from 28 mice). Together, these data show that PFs co-activated by a sensory stimulus are also likely to be aggregated in space.

**Figure 3:**
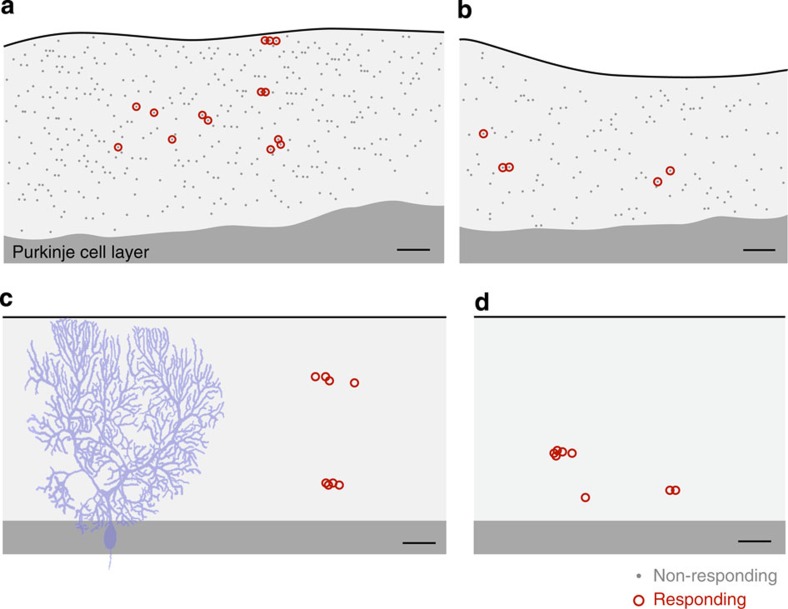
Maps of clustered parallel fibre activation in response to sensory stimulation. Spatial distribution of parallel fibres responding to sensory stimulation as determined from the high-resolution stacks and functional imaging data. Scale bars, 25 μm. Each map is from a different animal. (**a**,**b**) Spatial distribution of parallel fibres responding to sensory stimulation (red circles). Parallel fibres labelled with OGB-1, but not responding to the stimulus are marked as grey dots. (**c**,**d**) Data from two further experiments in which the non-responding fibres were not mapped. The distance from the pial surface as well as the fibres relative to each other was measured. A reconstructed Purkinje cell is shown for scale in **c**. See Supplementary Table 1 for further details and statistical analysis.

Finally, maps of sensory-activated PFs were constructed over large areas of the cerebellar molecular layer using the iterative high-resolution images acquired from contiguous regions of the molecular layer while delivering the same sensory stimulus in the course of the above experiments. These maps revealed multiple discrete clusters of responding fibres distributed across the molecular layer (Fig. 3). Statistical analysis of these maps confirmed that active fibres exhibited significant clustering (*P*<0.05 level for 11 out of 13 maps, Monte Carlo test, see Methods; for individual *P*-values see Supplementary Table 1).

### Exploring mechanisms underlying clustered activity

**Figure 4:**
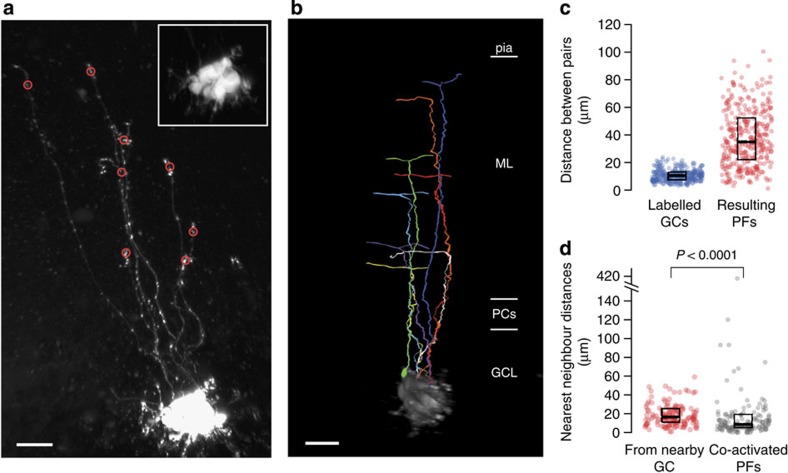
Neighbouring granule cells give rise to sparsely distributed parallel fibres. (**a**) Two-photon image of a group of 8 GCs labelled with a fluorescent dye *in vitro* (maximum intensity projection; to compensate for the high dynamic range, the square root of intensity is displayed). Inset: maximum intensity projection adjusted in brightness to clearly show the clustered GC somata. Note the highly divergent parallel fibre bifurcation points (red circles) in the molecular layer. Scale bar, 10 μm. (**b**) Three-dimensional reconstruction of the ascending axons and parallel fibre segments of the cells shown in **a**, superimposed on the image of labelled granule cell bodies. Scale bar, 10 μm. (**c**) Distances between labelled, neighbouring granule cells over all experiments (blue, left) and the distances between the resulting parallel fibre pairs (red, right). Median and interquartile range are indicated by the box plots (*n*=311 pairs). (**d**) Comparison of the nearest neighbour distances for parallel fibres arising from neighbouring granule cells (red, left) and co-active parallel fibres. Median and interquartile ranges are indicated by the box plots (both *n*=129 fibres). Note that fibres arising from neighbouring granule cells are significantly further spread than the co-active fibres found *in vivo* (two-tailed Mann–Whitney test; *P*<0.0001).

What are the circuit mechanisms underlying spatially clustered activity in PFs? One possibility is that a single mossy fibre bouton activates a cluster of granule cells, in turn giving rise to a cluster of active PFs. We used morphological analysis to evaluate the possibility that neighbouring GCs can drive co-activation of neighbouring PFs. Labelling a small cluster of neighbouring granule cells in cerebellar slices (Fig. 4a,b) resulted in labelled PFs with a broad distribution across the vertical depth of the molecular layer: on average, labelled PFs spread across 67% of the vertical extent of the molecular layer (s.d.=16.6%; *n*=24 clusters of 4–8 GCs in 9 slices from 5 mice). Indeed, the distances between the labelled PFs (median=35.0 μm, *n*=311 PFs in 9 slices from 5 mice) were significantly larger than the distances between densely packed granule cells giving rise to them (median=10.4 μm, *n*=255 identified GCs in 9 slices from 5 mice; *P*<0.0001, Mann–Whitney test; Fig. 4c). We next analysed the spatial distribution of axonal bifurcation points (that is, where the PF is formed from the granule cell ascending axon) using a nearest neighbour distance-based Monte Carlo test^35^. Of the 12 clusters of granule cells giving rise to 6 or more PFs, none exhibited statistically significant clustering of the bifurcation points in the molecular layer (Supplementary Table 2). Next, we compared the nearest neighbour distances of PFs arising from labelled granule cell clusters (indicated by red circles in Fig. 4a) to the nearest neighbour distances of co-active PFs *in vivo* (determined from reconstructed maps as shown in Fig. 3). With a median distance of 8.7 μm (interquartile range=4.8–18.8 μm, *n*=129 fibres in 15 mice), co-activated PFs were nearly twice as tightly spaced as PFs arising from neighbouring granule cells, which exhibited a median separation of 16.4 μm (Fig. 4d, interquartile range=10.4–24.7 μm, *n*=129 fibres in 24 clusters of 4–8 PFs in 9 slices from 5 mice; *P*<10^−4^, two-tailed Mann–Whitney test). Thus, PFs arising from immediately neighbouring granule cells exhibit much greater physical separation than the co-activated fibres we observe. Taken together, these data suggest that clustered PF activation is unlikely to be explained primarily by activation of a local granule cell cluster.

**Figure 5:**
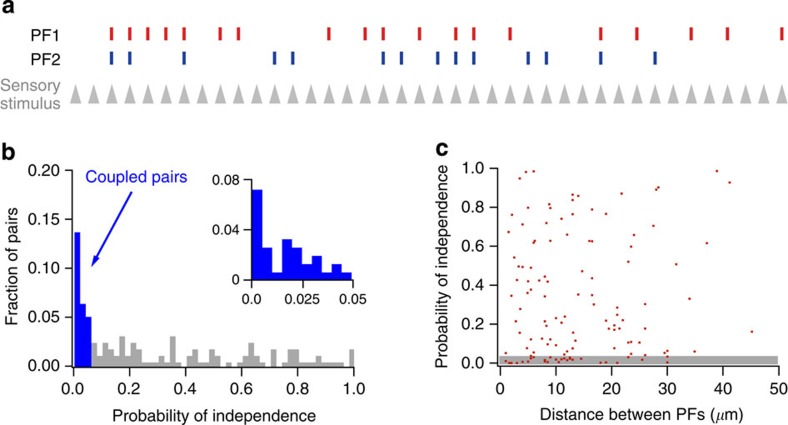
Correlation of response variability in neighbouring parallel fibres. (**a**) Raster plot of responses to a sensory stimulus for two neighbouring parallel fibres (stimulus frequency, 1 Hz). (**b**) Histogram of correlation in response variability across multiple trials for 126 pairs of neighbouring parallel fibres; coupled pairs are depicted by blue bars. The inset shows the significantly coupled part of the distribution at higher resolution. (**c**) Lack of correlation of independence probability and distance between pairs of parallel fibres (Spearman’s *r*=0.11; *P*=0.29, *n*=126 parallel fibre pairs from 28 animals). Note that coupled (highlighted with grey bar, *n*=34) and independent pairs (*n*=92) are not distributed differently (*P*=0.2645, Mann–Whitney test).

If the granule cells driving clusters of PFs are spatially dispersed, they can be driven either by separate mossy fibre inputs that are concurrently active or by the same mossy fibre providing collaterals innervating the parent granule cells. To investigate these possibilities, we analysed correlations of response variability (the variability between fibres in sequence of successes and failures over repeated stimulus presentations) to estimate the fraction of co-active fibres that are driven by common presynaptic input (Fig. 5). This revealed that ~22% of co-active PFs displayed significant correlations in their trial-to-trial variability (two-sided *P*<0.05, *n*=28 of 126 PF pairs from 28 mice). These correlated pairs represent co-active PFs that were likely to have been driven by at least one common mossy fibre, given that single mossy fibres can excite granule cells with sensory stimulation^33^. The remaining 78% of co-activated pairs were not significantly correlated, and thus are likely to be driven by different mossy fibres. These non-correlated PFs are nevertheless significantly clustered in space (median distance between ‘independent’ fibres=12.5 μm; median distance between labelled fibres=17 μm: *P*=0.0012; Mann–Whitney test, two-tailed). These data indicate that coactivity can result from activation by a common mossy fibre, but is predominantly evoked by distinct mossy fibres that are activated by the same stimulus.

## Discussion

We have provided a direct readout of an axonal spatial population code on the micron scale by imaging the activity across multiple neighbouring axons of a well-defined input population, the cerebellar PFs. Our results show that PFs activated by a physiologically relevant sensory stimulus exhibit clustered activation on the micron scale. This functional clustering of input is likely to have important implications for information transmission by PFs to the downstream Purkinje cells.

We developed an approach that enables the direct readout of activity from single and multiple neighbouring PFs *in vivo* using two-photon imaging. We demonstrate that individual PFs exhibit very low rates of activity at rest, but respond with bursts of action potentials to a brief sensory stimulus, consistent with electrophysiological measurements^36^. When examining activity across multiple neighbouring PFs—which is currently impossible using electrophysiological techniques—we found that PFs responding to a given sensory stimulus are present in the same imaging window at a significantly higher rate than predicted for randomly distributed fibres. Furthermore, within an imaging window, co-activated PFs were found to be significantly more closely spaced than randomly distributed fibres. Taken together, our results argue for a clustered co-activation of neighbouring presynaptic inputs driven by sensory stimulation.

Before bifurcating and forming PFs, granule cell axons ascend from the granular layer past the Purkinje cells into the molecular layer. In contrast to PFs, a single ascending axon can give rise to many (possibly dozens of) synapses with a given Purkinje cell^37^. This may lead to different spatial patterning of ascending axon synapses and PF synapses, which will need to be addressed in future studies.

Although previous studies have imaged axonal Ca^2+^ transients *in vivo* as a readout of projection patterns^25^,^26^, these studies did not directly examine the spatiotemporal pattern of activity relevant for postsynaptic integration. To our knowledge, the work presented here is the first time presynaptic activity has been spatially analysed with individual axon resolution in a mammalian system *in vivo*.

A complementary approach to mapping input distributions is to image postsynaptic calcium signals in individual spines *in vivo*. The only direct measurements of sensory-evoked spine activation *in vivo* thus far have failed to provide evidence for local clustered activation in cortical pyramidal neurons^17^,^18^. However, a considerable body of indirect evidence is consistent with clustered synaptic input *in vivo*^20^,^21^,^22^,^23^,^24^. Further work is required to establish whether clustered axonal activity is a general feature of sensory processing.

We investigated two potential circuit mechanisms that could give rise to spatially clustered PF activity. First, clusters of granule cells could be activated by a single mossy fibre bouton and give rise to clustered activity in PFs. This would require neighbouring granule cells to give rise to closely spaced PFs. Second, spatially dispersed granule cells could be activated either by different boutons of the same mossy fibre or by distinct mossy fibres responding to the same stimulus. This would require a mechanism for granule cells activated by the same sensory input to give rise to neighbouring PFs.

In our anatomical analysis of PFs arising from immediately adjacent granule cells, we found no significant tendency for these PFs to be clustered in the molecular layer, strongly arguing against the first possibility. This is consistent with previous studies that found no tendency for granule cell clusters to give rise to PF clusters^38^, as well as with more recent results that found granule cells arising from the same progenitor cells to give rise to closely spaced PFs, while their cell bodies are distributed over the whole depth of the granule cell layer^39^,^40^.

Instead, our data support the second possibility, which requires coordinated activation of spatially distributed granule cells giving rise to clusters of co-activated PFs. Such a circuit organization could, for example, be established by a developmental mechanism such as the one proposed by Espinosa and Luo^39^, where granule cells born from the same progenitor migrate together and form adjacent PFs. When the cell bodies come to rest in the granule cell layer, they form synapses with mossy fibres that are also just sprouting into the cerebellar cortex. The staggered arrival times of mossy fibres allow connections with simultaneously arriving granule cells to be formed in such a manner that mossy fibres conveying similar sensory inputs form synapses with granule cells giving rise to clustered PFs.

The highly ordered arrangement of the cerebellar cortical microcircuit, in conjunction with the physiological properties of the Purkinje cells and the synapses made by PFs onto their dendrites, allows the clustered activity we have observed to be read out by the downstream neurons in an efficient manner (see also Supplementary Discussion). First, PFs are orthogonal to the Purkinje cell dendritic tree, and the dense dendritic arborisation of the Purkinje cell ensures that there is a high anatomical connection probability (~0.5) between PFs and Purkinje cells^41^. Second, the reliable propagation of activity along the PF axon we have observed *in vivo*, together with the maintained proximity of neighbouring PFs over their considerable length (up to ~4 mm), suggests that many hundreds of Purkinje cells will be influenced by the same pattern of clustered activity. Third, synaptic integration of PF inputs in Purkinje cell dendrites has been shown to be highly sensitive to their spatial distribution: clustered PF inputs are efficient triggers of dendritic spikes^42^, dendritic calcium signals^43^,^44^,^45^ and cannabinoid release^42^,^46^. Finally, clustered PF synaptic input is a potent stimulus for synaptic plasticity at the PF-Purkinje cell synapse^45^,^46^,^47^, providing a mechanism for storage of clustered activity patterns. Taken together, our results suggest that the clustered spatial pattern of PF activity that we have observed should significantly enhance information transmission and storage driven by sensory activation of the PF pathway. They also contradict the longstanding theoretical prediction of scattered PF activation^27^, and should thus provide important constraints for any future models of cerebellar function.

## Methods

### Animals and surgery

All procedures were approved by the local ethical review committee and performed under license from the UK Home Office in accordance with the Animals (Scientific Procedures) Act 1986. Five- to nine-week-old female C57BL6/J mice (Harlan, UK) were anaesthetized using either injection or inhalation anaesthesia. Injection anaesthesia was achieved with an intraperitoneal (IP) injection of a ketamine-xylazine mixture (80–150 mg kg^−1^ ketamine and 12–18 mg kg^−1^ xylazine). Depth of anaesthesia was monitored by withdrawal reflex following foot pinch and anaesthesia boosted with ketamine (20–40 mg kg^−1^) as necessary. Inhalation anaesthesia was induced with 5% isoflurane in O_2_, supplemented with IP injection of chlorprothixene (1.0 mg kg^−1^) and maintained at 2% isoflurane in O_2_ during surgery. Dexamethasone (1–2 mg kg^−1^) was injected IP to reduce swelling of the brain and analgesics were given as needed. Care was taken to keep the animal’s temperature stable at 36.5–37.5 °C at all times using a homeothermic heating mat.

The crus II region was identified by location—roughly 3.5 mm lateral and 1 mm posterior of the interparietal-occipital bone fissure—and by the characteristic bone topology covering this region. To maximize chances of obtaining input from the perioral region, we targeted a central and very anterior part of the crus II folium^48^. A custom-made polycarbonate chamber^49^ was glued into place using superglue and additionally fixed into place using dental cement (Jet Denture Repair Acrylic, Lang Dental). Once the glue had dried, the bone was kept saturated with external saline (in mM: 150 NaCl, 2.5 KCl, 10 HEPES, 2 CaCl_2_, 1 MgCl_2_; pH 7.3).

Using a dental drill with a super fine diamond burr (Heraeus), a craniotomy was performed. While milling away the bone and when lifting the bone flap with forceps, the bone was kept damp and special care was taken to avoid any damage to the dura.

### Slice preparation

Parasagittal slices (200 μm) of postnatal day P24—27 mouse cerebellum were made using standard techniques^50^. Briefly, mice were decapitated under isoflurane anaesthesia, cerebella rapidly removed and transferred to ice-cold artificial cerebrospinal fluid (ACSF; 125 mM NaCl, 2.5 mM KCl, 26 mM NaHCO_3_, 1.25 mM NaH_2_PO_4_, 25 mM glucose, 1 mM MgCl_2_ and 2 mM CaCl_2_, bubbled with 5% carbon dioxide and 95% oxygen). Slices were cut with a vibrotome (Leica), incubated in ACSF at 35 °C for 30–45 min and then held at room temperature. During the experiment slices were continuously superfused with ACSF. Slice experiments were performed at room temperature.

### Two-photon microscopy

Two-photon imaging^51^ was performed using an *in vivo* optimized two-photon microscope with a low magnification (× 16), high numerical aperture (0.8) objective. Two-photon excitation was provided by a pulsed Ti:Sa laser (MaiTai HP, Newport), tuned to 810 nm. Fluorescence was detected with a photomultiplier tube (H-7422-P-40-MOD, Hamamatsu). Data were acquired using ScanImage 3.5 and 3.7.1 (ref. 52). To allow simultaneous imaging of PFs at different depths, the back-aperture of the objective was slightly underfilled, which also made the imaging experiments less sensitive to axial movement of the tissue. Slice experiments were performed on a separate two-photon microscope optimized for *in vitro* experiments.

### *In vivo* labelling of parallel fibres

Parallel fibres were labelled using bulk electroporation based on Nagayama *et al*.^29^ Guided by two-photon imaging, a pipette filled with a 5 mM solution of Orgeon-Green-488-BAPTA-1 (Invitrogen) in sterile water was positioned in the craniotomy, just above the dura. The pipette resistance was 1–1.5 MΩ when filled with this solution. Care was taken to expel as little dye as possible at this stage of the process by applying low pressure to the pipette at all stages of labelling: no more than 100 mbar for dura penetration and no more than 30 mbar during movement of the pipette through tissue. Using the negative fluorescence (‘shadow’) of the dye exiting the pipette tip to support orientation^53^,^54^, the pipette was diagonally advanced to the Purkinje cell layer and then further advanced into the granule cell layer until reaching a depth of 50–150 μm beneath the Purkinje cells, making sure the pipette tip remained clearly visible and unclogged. If necessary a brief pressure pulse of less than 100 mbar was used to unblock clogged pipettes. Once positioned, a train of 30 current pulses (1 ms, −30 μA, 2 Hz) was applied using a constant current stimulator triggered by an ITC-18-USB (HEKA) controlled by custom software written in Igor Pro (v6.1, Wavemetrics). After the current pulse train, the pipette was moved to the next location, taking care to limit the lateral movement to <100 μm. This was repeated to reach a total of six to eight spots. At each location, a spherical volume of cells ~60 μm in diameter was labelled. Because of the diagonal movement of the pipette, the labelled spots were located at varying depths spanning most of the granule cell layer within a single experiment. Following the labelling, the animal was left to rest under lighter anaesthesia (1.0% isoflurane) for at least 2 h, so that the extracellular dye could disperse and the intracellular dye could equilibrate along the axons of the labelled cells. The resulting labelled PFs were found to be randomly distributed with a slight, but in most cases (8 out of 11 stained regions), significant tendency towards regular spacing (see ‘Image analysis’ and ‘Statistics’ below).

### *In vivo* imaging

To improve the quality of imaging and minimize movement due to breathing and pulsation, the dura was removed, the brain covered with 1.5% low-temperature melting agar (Type III Agarose) in external saline, and topped off with a #1 coverslip, which was glued to the imaging chamber. To limit pulsation, care was taken to apply slight pressure to the brain without depressing it below the natural level. In experiments using inhalation anaesthesia, the anaesthesia level was then lowered to a level at which the mouse just remained quiescent during application of sensory stimulation (0.25–0.6% isoflurane in O_2_). In a separate group of experiments (*n*=5 mice), we performed electroencephalography (EEG) recordings and confirmed that animals held at this level of anaesthesia showed EEG activity that was clearly different from deep anaesthesia (see Supplementary Note 3 for discussion of the effect of anaesthesia type on the physiological measurements). Imaging was performed at least 100 μm distal to the electroporation site.

The spontaneously active fraction of PFs was determined by initially acquiring a stack (60 × 60 × 180 μm^3^) and then randomly selecting single fibres from this stack that were subsequently imaged at higher temporal resolution. Evoked activity in populations of PFs was imaged using a non-square frame scan (56 × 14 μm^2^) at 32 Hz. Linescans were acquired at 1 kHz, scanning a ~6 μm line perpendicular to the PF and centred on the bouton of interest. Both stimuli and the imaging system were triggered by an Arduino microcontroller board (http:// www.arduino.cc).

To verify that non-responding and silent fibres were capable of reporting activity, we locally applied high-K^+^ ACSF or electrically stimulated groups of PFs using a bipolar tungsten electrode (Harvard Apparatus Ltd.) diagonally lowered into the molecular layer in conjunction with a constant current stimulator (0.1 ms pulses, 10–70 μA). Both methods produced reliable Ca^2+^ signals in nearly all labelled fibres (99% and 91% of fibres responded; *n*=78 and 372, for high [K^+^] or electrical stimulation, respectively).

### *In vitro* labelling of PFs

Visually identified cerebellar granule cells were filled by single-cell electroporation^49^ using a 1,000-ms train of 500 μs, −6 V pulses applied at 50 Hz (Axoporator 800; Molecular Devices). Micropipettes (10–14 MΩ) were filled with a 1 mM solution of Atto-594 (Attotech GmbH) in saline (in mM: 150 NaCl, 2.5 KCl, 10 HEPES, 2 CaCl_2_, 1 MgCl_2_; pH=7.3). Groups of four to eight directly neighbouring granule cells were targeted. Following electroporation, at least 40 min were allowed for dye diffusion along the ascending axon and initial PF segment.

### Sensory stimulation

For sensory stimulation, 70 ms airpuffs to the ipsilateral perioral region were delivered using a Picospritzer II (Parker) through a glass capillary positioned in close proximity to the target region. Low pressure (median=20 p.s.i.) was used in order to ensure focal stimulation. Stimuli were repeated either 40 times with a 5-s interstimulus interval or 200 times with a 1-s interstimulus interval.

### Patch-clamp recording

Recordings from Purkinje cells *in vivo* were performed using standard methods^53^. Briefly, pipettes were filled with an internal solution containing (in mM): K-methanesulfonate 133, KCl 7, HEPES 10, Mg-ATP 2, Na_2_ATP 2, Na_2_GTP 0.5, EGTA 0.05, 0.1 Alexa Fluor 488; pH 7.2. Purkinje cells were identified by depth (>150 μm) and their distinct shape in the ‘shadow image’ (two-photon excitation performed at 910 nm). Data were low-pass filtered at 4 kHz and acquired at 20 kHz using an ITC-18 digitizer (Instrutech) controlled by AxoGraph X (http://www.axographx.com/). Analysis was performed using Igor Pro (Wavemetrics).

### EEG recording and analysis

A small (~1 mm) craniotomy was performed over the left neocortical hemisphere. A bipolar electrode consisting of two Teflon-coated silver wires (one 5 mm longer than the other) was inserted into the brain until the shorter wire came to rest on the dura. The electrode was fixed into position with a drop of super glue. Dental cement (Jet Denture Repair Acrylic, Lang Dental) was used to further stabilize the electrode. The EEG was recorded with an AC biopotential amplifier (P511 AC, Grass Technologies) and digitized with an ITC-18 (Heka). Traces were recorded for 60 s at a given anaesthesia concentration, with 8–10 min equilibration time between setting a new concentration and the subsequent recording. Traces were analysed using routines written in Python (http:// www.python.org) using Numpy (http:// www.scipy.org) and Matplotlib (http://matplotlib.sourceforge.net).

### Image analysis

Images were analysed using ImageJ (http://rsbweb.nih.gov/ij/), FIJI (http://fiji.sc/) and Igor Pro (Wavemetrics). Individual PFs were identified morphologically based on averaged images of the functional imaging stacks. In case of ambiguity, identity was verified using a higher resolution z-stack of the region. In the rare instances that fibres could not be clearly distinguished from other fibres they were excluded from analysis. Fluorescence traces corresponding to the selected regions of interest were extracted using ImageJ. If necessary, long traces (>30 s) were high-pass filtered in Fourier space, before further analysis. Event detection for spontaneous activity was performed in Igor Pro using a varying baseline thresholding routine, kindly provided by Taro Ishikawa (https://sites.google.com/site/tarotoolsregister/). Responses to sensory stimuli were determined using a semi-automated approach: transients showing a near instantaneous rise and slow decay were considered to be responses if they occurred time-locked to the sensory stimulation and exceeded a level of two standard deviations above baseline. Similarly, a fibre was considered responsive to a given stimulus if the stimulus-triggered average showed a significant (>2 s.d. over baseline) transient increase time-locked to the stimulus. Fibres responding to the same stimulus were termed ‘co-activated’. Event amplitudes were measured relative to the baseline immediately preceding event onset. For linescans Ca^2+^ transients were quantified using standard methods (‘dF over F_0_’)^55^.

SNRs were determined from active/responding fibres acquired at the same frame rate as the population imaging data. The SNR of events was calculated as the ratio between the peak amplitude and the root mean square of the baseline before the event. To allow comparison between experiments and pooled analysis of all SNRs, the amplitudes of both noise and events were normalized to the mean event amplitude for a given fibre. This resulted in different scaling of the noise peaks for spontaneous and evoked plots, because of the larger amplitudes of the evoked events.

Maps of responding PFs were generated from the functional imaging t-stacks using the 3D coordinates of the respective FOV within the labelled region. In some experiments, high-resolution z-stacks of the whole region subjected to functional imaging were acquired and used to generate a map of the labelled fibres, which was then registered with the activity map.

Labelling density and labelling pattern were determined using a high-resolution XYZ-stack. XZ-images (each a sum of three neighbouring XZ slices, corresponding to a 1-μm-thick XZ-section) were extracted and the coordinates of all fluorescent spots (verified to correspond exclusively to single PF boutons using the full stack) were determined. The resulting coordinate files were subjected to spatial patterning analysis (see below). The density of labelled PF boutons was then multiplied by the average distance between two boutons on the same fibres (3.7 μm in the imaged region^37^), to estimate the density of labelled fibres (making use of the fact that the examined sections extended 1 μm along the PFs). Finally, the labelled fraction was estimated by dividing the density of labelled fibres by the known density of PFs (5–6 per μm^2^) (ref. 56). The resulting value was checked for plausibility by determining the number of labelled PFs within the area of a single Purkinje cell dendritic tree (~200 μm width in our mice line). This resulted in 540±44 PFs (*n*=12 mice), not significantly different from the 570 PFs expected for our estimated labelling density (0.38% of 150,000; *P*=0.56, *t*-test). Two-dimensional spatial patterning analysis was performed using the Monte Carlo test described below. In some cases, structural images with a large intensity range are displayed as square root of the intensity to allow clear visualization of bright and dim regions in a single image, as noted in the figure legend.

### Statistics

Statistics were performed using Igor Pro (Wavemetrics), InStat 3 and Prism 6 (both: GraphPad Software), Python (www.python.org), Numpy and Scipy (both: www.scipy.org). *P*-values smaller than 0.05 were considered significant.

To determine the likelihood of parallel fibres having common presynaptic input, Pearson’s correlation coefficients as well as the corresponding *P*-values were calculated for response raster plots of each pair. The resulting *P*-value was used as ‘probability of independence’ for the respective pair. If that probability was below 0.05, the pair was considered significantly coupled. As this analysis requires simultaneously imaged PFs, it was limited to pairs contained within a field of view.

Spatial distributions of parallel fibres were tested for significant deviation from complete spatial randomness using a Monte Carlo test. Briefly, 10,000 random distributions of points (sample size equal to the respective experimental sample) were generated and the average nearest neighbour distance for each of these random samples calculated. The average nearest neighbour distance of the experimental sample was then compared with the distribution obtained for the random samples and both the direction and significance of the deviation from randomness determined. Shorter nearest neighbour distances indicate a tendency towards clustering, whereas longer distances indicate a tendency towards regular spacing. The reported *P*-values are two-sided and indicated if this tendency was a significant deviation from random. The area for the simulations was selected using either the stained and imaged area (for the *in vivo* experiments) or the bounding width of labelled fibres multiplied by the thickness of the molecular layer (for the slice experiments). In the latter case, we verified that this selection of area size did not prevent us from detecting clustering by repeating the simulations using the doubled area. Even under these extreme conditions none of the experiments showed significant clustering of labelled bifurcation points.

## Additional information

**How to cite this article:** Wilms, C. D. and Häusser, M. Reading out a spatiotemporal population code by imaging neighbouring parallel fibre axons *in vivo*. *Nat. Commun.* 6:6464 doi: 10.1038/ncomms7464 (2015).

## Supplementary Material

Supplementary Information Supplementary Figures 1-4, Supplementary Tables 1-3, Supplementary Notes 1-3, Supplementary Discussion and Supplementary References
