# Supplementary material for: Reading out a spatiotemporal population code by imaging neighbouring parallel fibre axons in vivo
Source: Nat Commun. 2015 Mar 9;6:6464. doi: 10.1038/ncomms7464 (PMC4366501; doi:10.1038/ncomms7464)
Supplement: Supplementary Information — Supplementary Figures 1-4, Supplementary Tables 1-3, Supplementary Notes 1-3, Supplementary Discussion and Supplementary References [file ncomms7464-s1.pdf]

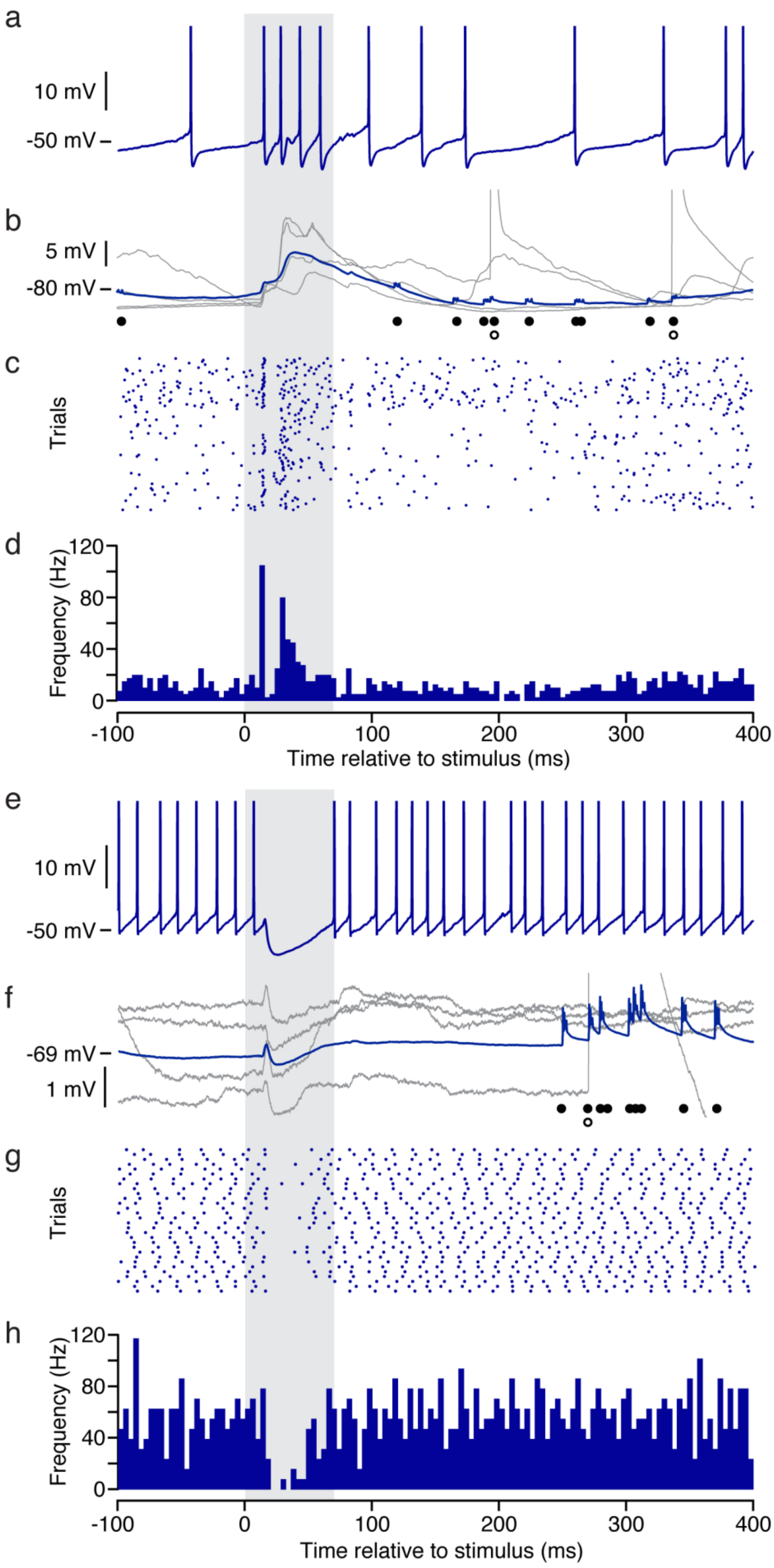

a
10 mV
-50 mV
b
5 mV
-80 mV
c
Trials
d
Frequency (Hz)
120
80
40
0
-100
0
100
200
300
400
Time relative to stimulus (ms)
e
10 mV
-50 mV
f
-69 mV
1 mV
g
Trials
h
Frequency (Hz)
120
80
40
0
-100
0
100
200
300
400
Time relative to stimulus (ms)

Supplementary Figure 1: Purkinje cell responses to sensory stimulation.

Whole-cell patch-clamp recordings from Purkinje cells in crus II *in vivo* showing subthreshold and suprathreshold responses to perioral sensory stimulation (grey bar). Recordings from two Purkinje cells are shown; **a-d** shows a recording dominated by excitation, and **e-h** a recording dominated by inhibition. **a and e**: Recording from a Purkinje cell in the absence of holding current showing spontaneous spiking and a sensory-evoked changes in spiking (spikes are clipped). **b and f**: Subthreshold responses to the same stimulus recorded at a hyperpolarized membrane potential. The averages of 20 (b) and 90 (f) traces are shown in blue with four representative traces in grey. Complex spikes are indicated below by a filled black circle (for the average trace) and open circles (for example traces; spikes are clipped). Traces containing a complex spike within ±100 ms of the stimulus onset were not included in the average. **c and g**: Raster plot of all simple spikes across all trials (arranged vertically). **d and h**: Corresponding post-stimulus time histogram (4 ms bins).

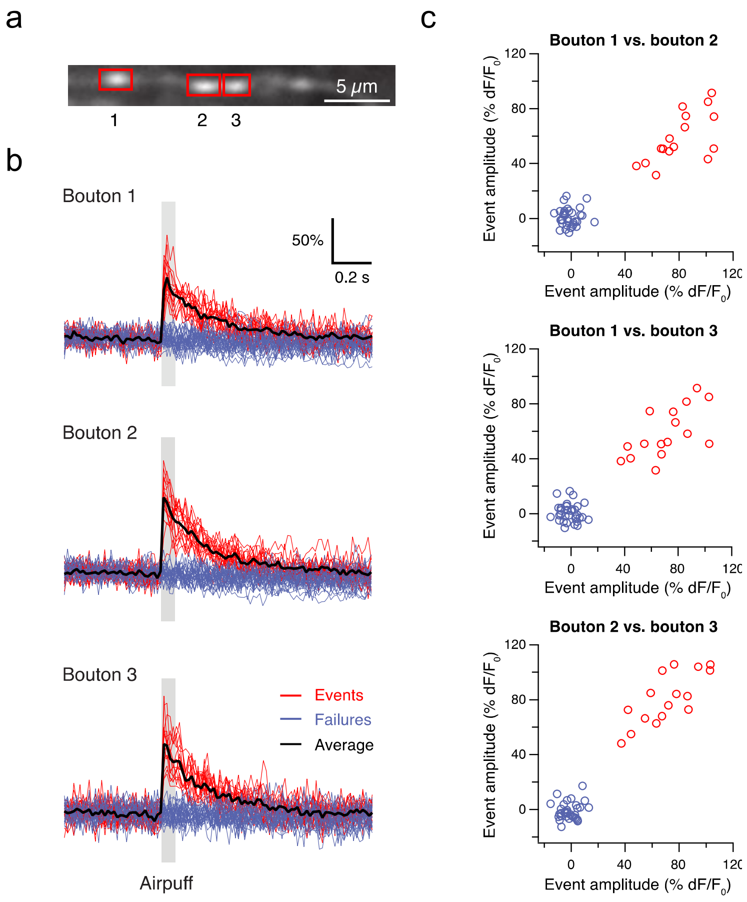

Supplementary Figure 2: Sensory-evoked calcium transients measured along a single parallel fibre axon*.*

(**A)** Two-photon image of a single parallel fibre axon *in vivo*. (**B**) Calcium transients evoked by a sensory stimulus (airpuff to the perioral region) were measured in the three putative boutons (red ROIs) along the same parallel fibre. Trials exhibiting responses are shown in red, failures in blue, and the average of the responses is shown in black. (**C**) Plotting the peak response amplitudes of one bouton against another shows that the response amplitudes in all three boutons are highly correlated, with events and failures occurring in a coordinated manner across all boutons (for all combinations $R \geq 0.93$, $P < 10^{-10}$). Note also the clear separation between failures and responses, indicating the high signal-to-noise of the measurements.

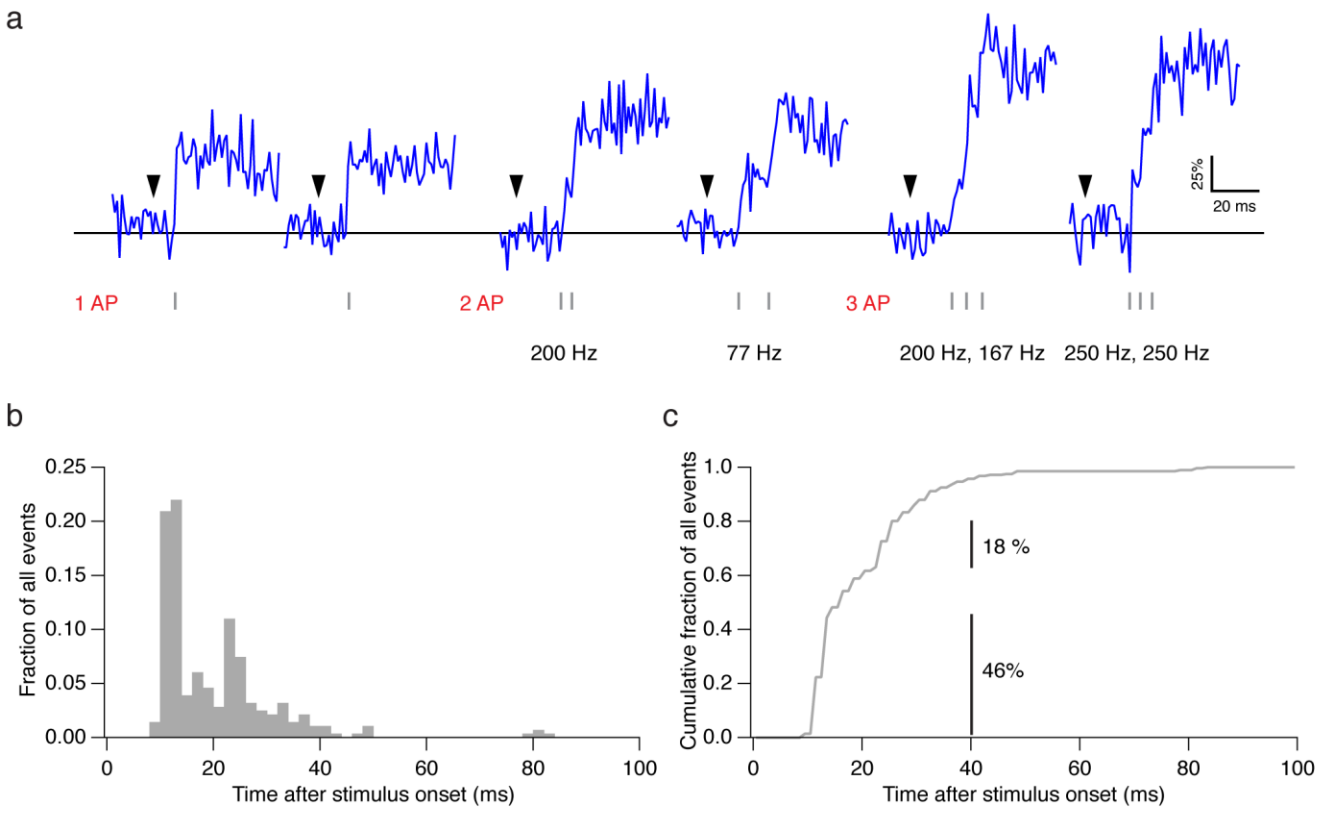

Supplementary Figure 3: Estimating action potential rates and timing from presynaptic calcium transients *in vivo*.

a: Representative sensory-evoked calcium signals recorded from single parallel fibre boutons *in vivo*. Note the clear shoulders in the rising phases of individual responses, as well as the progressive increase in overall transient amplitude with increasing number of action potentials. Responses have been ordered by the number of estimated underlying action potentials. Arrowheads indicate stimulus onset. Vertical bars below the traces indicate the timing of the inferred action potentials. Where applicable, the instantaneous frequencies are provided below. These are in the range expected for granule cell responses to sensory stimuli[14, 15]. **b**: PSTH of PF responses with 2 ms bin width. Note the two clear peaks at 10 and 22 ms. **c**: cumulative histogram of the same data (n = 23 PFs from 11 mice).

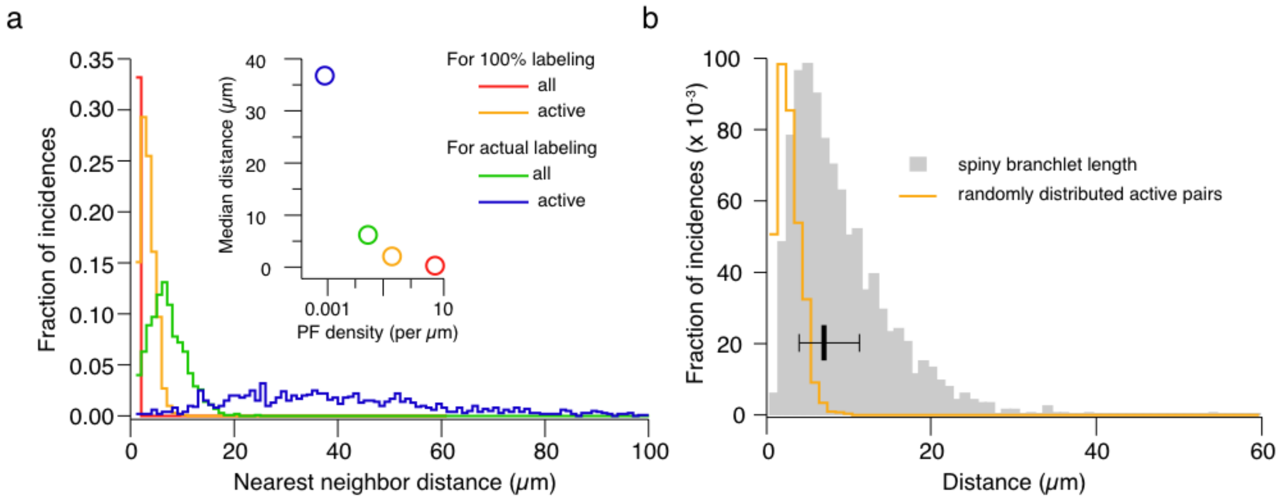

Supplementary Figure 4: Estimating the density of all active PFs.

a: Nearest neighbour distributions for 1000 PFs at the density of our labelling (green) and at 2.8% of the labelling density (blue, corresponding to the responding fraction we find, but randomly distributed), as well as the nearest neighbour distribution for 1000 PFs at the native density of 6 per μm^2 (red) and at 2.8% of this density (orange). The median nearest neighbour distances for these four densities are plotted in the inset (same colour code). Further details in Supplementary Table 3 **b**: Comparing the extrapolated PF densities to the length distribution of spiny branchlets of Purkinje cell dendrites (grey). The nearest neighbour distance distribution calculated for 2.8% randomly distributed PFs at the actual PF density is plotted in orange (scaled to peak of branchlet length distribution). The median and interquartile range of the spiny branchlet length is shown in the overlaid whisker plot.

Supplementary Table 1: Spatial statistics of co-activated parallel fibres.

# of PFs	found distance	expected distance	difference (µm)	tendency	2-tailed *P*[1]
8	5.8 µm	23.2 µm	-17.4 µm	clustered	*0.0001*
8	7.5 µm	28.6 µm	-21.1 µm	clustered	*0.0001*
15	9.9 µm	30.0 µm	-20.2 µm	clustered	*0.0001*
19	11.9 µm	24.3 µm	-12.5 µm	clustered	*0.0001*
5	13.4 µm	49.4 µm	-36.0 µm	clustered	*0.0016*
7	17.3 µm	50.7 µm	-33.4 µm	clustered	*0.0018*
8	10.7 µm	23.0 µm	-12.3 µm	clustered	*0.0062*
8	14.1 µm	29.1 µm	-15.0 µm	clustered	*0.0108*
7	10.7 µm	29.3 µm	-18.6 µm	clustered	*0.0120*
6	11.9 µm	24.7 µm	-12.8 µm	clustered	*0.0298*
8	32.7 µm	59.0 µm	-26.3 µm	clustered	*0.0300*
6	34.7 µm	58.3 µm	-23.6 µm	clustered	*0.0958*
6	32.4 µm	47.4 µm	-15.0 µm	clustered	*0.2002*

Supplementary Table 2: Spatial statistics of parallel fibres arising from neighbouring granule cells.

# of PFs	found distance	expected distance	Difference	tendency	2-tailed *P*[1]
7	15.6 µm	16.6 µm	-1.0 µm	clustered	*0.719*
8	7.9 µm	8.4 µm	-0.5 µm	clustered	*0.801*
6	11.1 µm	11.4 µm	-0.4 µm	clustered	*0.897*
8	13.8 µm	13.7 µm	0.1 µm	regular	*0.967*
7	19.9 µm	19.3 µm	0.6 µm	regular	*0.897*
6	12.4 µm	11.7 µm	0.7 µm	regular	*0.857*
7	17.3 µm	15.8 µm	1.5 µm	regular	*0.857*
7	14.0 µm	12.7 µm	1.2 µm	regular	*0.683*
8	17.6 µm	16.2 µm	1.4 µm	regular	*0.671*
6	25.9 µm	22.0 µm	3.9 µm	regular	*0.486*
6	15.9 µm	13.4 µm	2.5 µm	regular	*0.448*
6	20.6 µm	14.3 µm	6.3 µm	regular	*0.090*

Supplementary Table 3: PF nearest neighbour distance as a function of PF density.

Scenario	PF density	Nearest neighbour distance
labelled PFs	2.28×10^{-2} per μm^2	6.2 µm
all PFs	6.0 per μm^2	0.4 µm
2.8% of labelled PFs	6.384×10^{-4} per μm^2	36.7 µm
2.8% of all PFs	0.168 per μm^2	2.2 µm

[1] Probability of finding the reported nearest neighbor distances assuming a random distribution

Supplementary Note 1: Responses of Purkinje cells to sensory stimulation

We performed whole-cell current-clamp and loose cell-attached recordings to assess the impact of our sensory stimuli on postsynaptic Purkinje cells *in vivo*. Purkinje cells exhibited both increases (Supplementary Figure 1a-d) and decreases (Supplementary Figure 1e-h) in spiking in response to sensory stimulation, in agreement with extracellular recordings from crus II Purkinje cells in rats[1]. This variability presumably reflects the location of the recorded Purkinje cells with respect to the active granule cell patches[1, 2]. Most cells (22/31 recordings) exhibited biphasic responses, with both increases and decreases in spiking, likely reflecting the combined recruitment of excitation and inhibition by sensory activation of mossy fibres. The Purkinje cells responding to the stimulus with an initial transient increase in spiking (24/31 recordings in 11 mice) showed an average peak response (as determined from 4 ms bin width PSTH constructed over 100 trials) of 96 ± 28 Hz with the strongest responding cell peaking at 155 Hz. While many cells respond to the airpuff stimulation with increases in spike rate, these increases were generally short-lived, with 8 of the 10 strongest-responding cells producing a peak in the PSTH of just one bin (at 4 ms bin width). This is also reflected in the low number of extra spikes introduced by the stimulation (median = 0.4 extra spikes; n = 24 recordings with an excitatory component in the PSTH, determined using baseline firing-corrected cumulative spike times as previously described[3]). Indeed, over the whole population Purkinje cells showed a stimulus evoked net *reduction* in spiking (median = -0.12 ‘extra spikes’). Thus, our sensory stimulus can trigger changes in spiking comparable to those previously observed with brief sensory stimulation[1, 4]. On the other hand, the activity levels evoked by our stimulus are much smaller than those reported in Purkinje cells during sensory-motor behaviour. During such behavioural tasks Purkinje cells can reach much higher firing frequencies (over 300 Hz have been reported[5-7]) than we found for an isolated stimulus with such behaviourally relevant increases lasting much longer (often > 100 ms[8-10]). This demonstrates that our stimulus is far from saturating Purkinje cell spiking responses.

In some experiments Purkinje cells were hyperpolarized (see Supplementary Figure 1b,f), revealing sensory-evoked EPSPs with a very large variability in amplitude. In most cases the PSP contained two distinct excitatory components, which were also reflected in the spiking responses. The timing of these components matches the timing of the two peaks in the PF event latencies (see Supplementary Figure 3b) and is consistent with the activation of trigeminal and corticopontine mossy fibre inputs projecting to crus II[11]. At less hyperpolarized potentials (~-65 mV), a strong inhibitory component was observed, which often terminated the excitatory component. Supplementary Figure 1f shows a representative recording, with the excitatory component being restricted to a FWHM of 3.3 ms by feed-forward inhibition. This inhibitory component is also reflected in the spiking pattern of non-hyperpolarized recordings, where 28 out of 31 cells showed a transient decrease in spiking, commonly following the initial increase (median number of dropped spikes = 0.5, n = 28 recordings; see Supplementary Figure 1g,h).

Supplementary Note 2: Estimating number and timing of APs underlying single PF responses

Linescans were used to estimate the number of action potentials underlying sensory-evoked Ca^{2+} transients in single parallel fibre boutons. Previous *in vitro* studies have shown that single action potentials in granule cells produce highly reliable Ca^{2+} transients in single parallel fibre boutons, and that the bouton Ca^{2+} transients provide an accurate readout of the number of granule cell action potentials[12, 13]. Using high-speed linescans (1 kHz) across single parallel fibre boutons *in vivo*, inspection of the rising phase of individual events allowed us to identify clear inflections characteristic of multiple action potentials (Supplementary Figure 2a). We found that 83% (n = 164/198 responses) of all sensory-evoked Ca^{2+} transients exhibited clear shoulders in the rising phase, implying multiple underlying action potentials. This is consistent with *in vivo* patch-clamp recording experiments from granule cells showing that sensory stimulation produces bursts consisting of variable numbers of action potentials[14]. To estimate action potential numbers, the rising phases were analysed in more detail: inflections were taken to be indicative of an underlying action potential if a nearly instantaneous rise above noise level was interrupted by at least 3 points with no further such rise (interpreted as the onset of transient decay). On average, we estimated that sensory-evoked responses consisted of 2.6 ± 0.9 (SD; n = 101 responses) action potentials, with an average frequency of 134 ± 73 Hz (SD, n = 191 APs).

Linescans also allowed the estimation of response latencies by comparing stimulus onset and response onset (see Supplementary Figure 2bc). We find that the latencies are bimodally distributed, with peaks at ~10 ms and ~22 ms. We find that 46% of PF responses occur within a 4 ms time window 10 ms after stimulus onset. A further 18% occur within a 4 ms time window with 22 ms latency. These two peaks are expected to correspond to the short latency trigeminal and longer latency corticopontine inputs to crus II[11]. Thus, the majority of inputs are active within two narrow time windows, allowing for efficient post-synaptic integration.

Supplementary Note 3: Effect of different forms of anaesthesia

Besides the difference in the number of spontaneously active PFs (see main text), we found that stimulus response rates were increased under light isoflurane anaesthesia (median response rate = 0.59, n = 58 fibres) when compared with ketamine-xylazine (median response rate 0.3, n = 136 fibres; p = 0.0004, two-sided Mann-Whitney).

In contrast, the clustering we find was not affected by the anaesthesia regime: The nearest neighbour distances between co-active fibres in the maps (e.g. Fig. 3) are not significantly different between KX and IF (two-tailed p = 0.8086, Mann-Whitney test, n = 21 pairs in 3 maps KX vs. 108 pairs in 13 maps IF, median NN distance = 8.0 µm vs. 8.8 µm), the cluster size in the activation maps is also not significantly different (two-tailed p = 0.1362, Mann-Whitney test, n = 19 clusters vs. 51 clusters, median cluster size = 2 for both). This is a strong indication for the robustness of our finding of clustered parallel fibre activation.

Supplementary Discussion

Functional relevance

For the interpretation of our results, it is important to consider that only ~0.4% of all PFs in the region are labelled in our preparation, implying that clusters will contain more responding fibres than the 2 – 5 that we have imaged and that the distances between co-active fibres will thus be shorter. To estimate how pronounced this latter effect is, we simulated fields of different fibre densities. To simulate the activation density we observed, we used the actual PF density[16]; our labelling density; or 2.8% of each of these, using 1000 fibres for each the four scenarios (see Supplementary Table 3). For the resulting fields of fibres we determined the nearest neighbour distance distributions and the median nearest neighbour distances (see Supplementary Figure 4a). We find that the median nearest neighbour distance is 16-fold smaller when going from our labelling density to the actual PF density, for both the 'all fibres' scenario and the randomly distributed active fibres scenario. We used randomly distributed active fibres in this simulation (to avoid making any assumptions about a mathematical model of clustering). As clustered points by definition have shorter nearest neighbour distances than randomly distributed points, this analysis provides a "worst case" upper limit for the distances between co-activated fibres. Given the strong decrease in nearest neighbour distance when going from our labelled fibre density to the actual fibre density, the distances between all co-activated fibres will therefore be significantly shorter than observed in our experiments.

When considering the postsynaptic consequences of clustered parallel fibre activity, a main determinant will be the spatial configuration of these inputs on the dendrites of the postsynaptic cell: i.e. do the clustered PFs target neighbouring spines, the same spiny branchlet, or different branchlets. To determine if the distances between co-active fibres that we extrapolate above are on a scale that would allow multiple fibres from a given cluster to target the same spiny branchlet, we extracted the spiny branchlet length distribution from a published, reconstructed rat Purkinje cell model[17]. We find that for randomly distributed active PFs (assuming 2.8% activation rate), over 95% of the nearest neighbour distances are shorter than the median spiny branchlet length (7.1 µm; orange trace in Supplementary Figure 4b). As clustered fibres will be even more densely packed, this suggests that the clustering we find is likely to be of relevance to synaptic integration in individual spiny branchlets of post-synaptic Purkinje cells.

Besides distance between fibres, the orientation of a PF pair relative to a spiny branchlet will be important in determining the postsynaptic effect of that fibre pair. To estimate the influence of PF-cluster to branchlet orientation, it is necessary to consider the space covered by spines from a single spiny branchlet in comparison to the distances between co-activate fibres. Based on their anatomical work, Fox & Barnard[18] found that given the dense packing of spines, a spiny branchlet roughly corresponds to a cylinder with a diameter of 3 µm on average. Comparing this to our extrapolated average nearest-neighbour distance of 2.2 µm (for random distribution, due to clustering this is expected to be significantly shorter), it is clear that while orientation will surely play a role, its effect on the readout of clustered PF input is expected to be small. This is all the more true for metabotropic signalling.

Importantly, each PF is predicted to synapse onto 135 PCs/mm (in rats[19]). As a consequence, there are a large number of target cells, each receiving inputs from a given PF cluster in a different post-synaptic spatial configuration. Together with the above density analysis, this clearly shows that the clustering we report here is sufficiently tight to allow co-active PFs to make contact with the same PC dendritic branchlet in multiple PCs along a beam, influencing the integration of PF input by PCs.

Supplementary References

1. Bower, J.M. & Woolston, D.C. *J Neurophysiol* **49**, 745-766 (1983).
2. Rokni, D., Llinas, R. & Yarom, Y. *Front Syst Neurosci* **1**, 1 (2007).
3. Mittmann, W., Koch, U. & Hausser, M. *J Physiol* **563**, 369-378 (2005).
4. Kunzle, H. *Anat Embryol (Berl)* **175**, 537-544 (1987).
5. Edgley, S.A. & Lidierth, M. *J Physiol* **401**, 399-415 (1988).
6. Krauzlis, R.J. & Lisberger, S.G. *J Neurophysiol* **72**, 2045-2050 (1994).
7. Medina, J.F. & Lisberger, S.G. *J Neurophysiol* **102**, 2039-2054 (2009).
8. Armstrong, D.M. & Edgley, S.A. *J Physiol* **352**, 403-424 (1984).
9. Berthier, N.E. & Moore, J.W. *Exp Brain Res* **63**, 341-350 (1986).
10. Thach, W.T. *J Neurophysiol* **33**, 537-547 (1970).
11. Morissette, J. & Bower, J.M. *Experimental Brain Research* **109**, 240-250 (1996).
12. Brenowitz, S.D. & Regehr, W.G. *Journal of Neuroscience* **27**, 7888 - 7898 (2007).
13. Zhang, W. & Linden, D.J. *Cerebellum* **11**, 121-131 (2012).
14. Chadderton, P., Margrie, T.W. & Hausser, M. *Nature* **428**, 856-860 (2004).
15. Jörntell, H. & Ekerot, C.F. *Journal of Neuroscience* **26**, 11786 - 11797 (2006).
16. Palay, S.L. & Chan-Palay, V. *Cerebellar cortex: cytology and organization* (Springer, Berlin, Heidelberg, New York,, 1974).
17. Roth, A. & Häusser, M. *J Physiol* **535**, 445-472 (2001).
18. Fox, C.A. & Barnard, J.W. *J Anat* **91**, 299-313 (1957).
19. Harvey, R.J. & Napper, R.M. *Prog Neurobiol* **36**, 437-463 (1991).
